# Major adverse cardiovascular events among patients with type-2 diabetes, a nationwide cohort study comparing primary metabolic and bariatric surgery to GLP-1 receptor agonist treatment

**DOI:** 10.1038/s41366-023-01254-z

**Published:** 2023-01-20

**Authors:** Erik Stenberg, Erik Näslund

**Affiliations:** 1 Department of Surgery, Faculty of Medicine and Health, Örebro University, Örebro, Sweden; 2 Division of Surgery, Department of Clinical Sciences, Danderyd Hospital, Karolinska Institutet, Stockholm, Sweden

**Keywords:** Obesity, Type 2 diabetes

## Abstract

**Background:**

Glucagon-like Peptide-1 receptor agonists (GLP-1 RA) and metabolic and bariatric surgery (MBS) both improve cardiovascular outcomes in patients with severe obesity and type-2 diabetes (T2D). The aim of the present study was to assess the impact of MBS on major cardiovascular adverse events (MACE) in patients with severe obesity and T2D compared to patients with T2D treated with GLP-1 RA.

**Subjects and methods:**

In this propensity score matched cohort study on nationwide data, patients with T2D and severe obesity who underwent MBS in Sweden from 2007 until 2019 were identified from the Scandinavian Obesity Surgery Registry and matched to a non-surgical group with T2D treated with GLP-1 RA (81.7% liraglutide, 9.0% dulaglutide, 6.0% exenatide, 1.6% lixisenatide and 0.8% semaglutide) from the general population using generalized linear model. Major outcome was MACE (hospitalization for acute coronary syndrome or cerebrovascular event or all-cause death), evaluated with multivariable Cox regression.

**Results:**

In total 2161 patients (obesity class I (10.2%), class II (40.3%), class III (49.5%)) were matched to 2161 non-surgical patients (mean age 51.1 ± 9.29 vs 51.5 ± 8.92 years, 64.8% vs. 64.4% women, with mean number of diabetes drugs of 2.5 ± 0.89 vs 2.6 ± 0.87, a mean duration of diabetes of 6.0 ± 4.15 vs 6.0 ± 4.51 years with 44.2% vs. 42.8% being treated with insulin at baseline). During the study period, 113 patients (8-year cumulative incidence 9.3%) compared to 130 non-surgical patients (8-year cumulative incidence 11.3%) suffered from MACE or all-cause mortality (HR 0.76, 95%CI 0.59–0.98), and 69 patients (8-year cumulative incidence 5.1%) compared to 92 non-surgical patients (8-year cumulative incidence 7.6%) suffered from a non-fatal MACE (HR 0.68, 95%CI 0.49–0.93).

**Conclusion:**

In this matched cohort study, MBS was associated with lower risk for MACE compared to treatment with early GLP-1 RA in patients with T2D.

## Introduction

Metabolic and bariatric surgery (MBS) has been found to have a significant protective effect on cardiovascular disease (CVD). Patients with a history of MBS have been shown to have a lower risk of mortality after a myocardial infarction (MI) than controls who have not undergone MBS. Furthermore, patients with cerebrovascular accidents have a lower risk of in hospital mortality if they have a history of previous MBS [1]. In patients with severe obesity and type 2 diabetes (T2D) MBS was associated with a lower risk for major adverse cardiovascular events (MACE) [2]. Two recent observational studies have also demonstrated that MBS may have a role in the secondary prevention of CVD. Patient with severe obesity and a previous MI who underwent MBS were found to have lower risk of death and a new MI than matched controls who did not undergo MBS [3]. Similarly, MBS was associated with a lower risk of MACE in patients with pre-existing CVD and severe obesity [4]. MBS has been found to induce remission of many risk factors for CVD such as T2D, hypertension and dyslipidaemia [5–7].

MBS is associated with increased postprandial circulation levels of incretin hormones such as glucagon-like peptide-1 [8, 9]. These postprandial changes have been proposed as one of several mechanisms for the weight loss and positive outcomes seen after MBS [10].

GLP-1 receptor agonists (RA) are used in the treatment of T2D. Liraglutide has been found to reduce weight and lowers the risk of MACE in patients with T2D and CVD regardless of prior MI or not [11]. Recently the dual agonist of GLP-1RA/glucose-dependent insulinotropic polypeptide (GIP) (Tirzepatide) has been found to improve glycaemic control and reduce weight in patients with T2D and severe obesity [12] and in patients with severe obesity to reduce weight by up to 20% after a 72 week study period [13]. Thus GLP-1 RA and MBS both improve CVD outcomes in patients with severe obesity and T2D.

The aim of this study was to assess the impact of MBS on CVD outcomes in patients with severe obesity and T2D compared to non-surgical patients with T2D treated with GLP-1 RA.

## Methods

The study was based on a matched cohort of data including all adult patients (≥18 years) with a registration in the Scandinavian Obesity Surgery Registry (SOReg) and matched (1:10) individuals from the general population with exact matching based on age, sex and county of residence. SOReg is a national research and quality registry which started in 2007 and currently covering virtually all bariatric and metabolic surgical procedures in Sweden. The registry is continuously validated and has so far been reported to have very high validity of data [14]. By use of personal identification numbers (unique to all residents in Sweden), the registry was linked to the Cause of Death registry, the Total Population registry, the National Patient registry, and the Prescribed Drugs registry. The Total Population registry covers all mortality with registrations of causes of death in the Cause of Death registry [15]. The national patient registry is a nationwide registry covering all hospital admissions since 1987, with an outpatient component which started in 2001 and currently covers >95% of outpatient visits in specialized health care [16]. The Prescribed Drugs registry was established in 2005 and includes all dispensed, prescribed drugs classified according to the World Health Organization Anatomic Therapeutic Chemical classification system (ATC) [17]. Socioeconomic data was based on data from the Longitudinal integrated database for health and insurance and labor market studies [18].

### Definitions

T2D was defined by a previous diagnosis of diabetes and treatment with at least one antidiabetic drug within 12 months of the intervention. Cardiovascular comorbidity was defined by a previous diagnosis of ischemic heart disease (ICD-10: I20-22), heart failure (ICD-10: I50) or arrhythmic heart disease (ICD-10: I47-48). Dyslipidaemia was defined by the use of lipid modifying drugs (ATC: C10). COPD was defined as admission for COPD or a complication of COPD with COPD as secondary diagnosis, or prescription of an anticholinergic drug (ATC: R03BB), long-acting beta-2 agonist (ATC: R03AC12-R03AC18) or a combination of these (ATC: R03AL), indicating moderate-to-severe COPD [19].

Disposable income (total taxable income minus taxes and negative transfers) was adjusted to the 2019 consumer price index and divided into percentiles (lowest 25th, 25th to median, median to 75th, and highest 75th) based on the disposable income of an age, sex and county of residence matched cohort of the normal population.

The highest level of education was divided in to three categories based on the highest completed education at the time of intervention: primary education (9 years of schooling), secondary education (completed 10–12 years of schooling) or higher education (completed college or university degree).

### Matching

From this cohort, patients with T2D with active pharmacological treatment were identified. Patients who underwent a primary Roux-en-Y gastric bypass or sleeve gastrectomy procedure and who were not treated with GLP-1 RA after surgery were matched to non-operated individuals with T2D who received GLP-1 RA as part of the diabetes treatment for a period of at least 2 years after initiation of the intervention. The matching was conducted using a 1:1 Propensity score match with a generalized linear model and a caliper of 0.2 including age at the time of intervention, sex, year of when the intervention started, county of residence, disposable income, highest level of education, cardiovascular comorbidity, COPD, dyslipidaemia, number of diabetes drugs, insulin treatment, and duration of diabetes.

### Outcomes

Main outcome was the occurrence of major cardiovascular event (MACE) which was defined as hospitalization for acute coronary syndrome (ICD-10: I20.0, I21-22) or cerebrovascular event (ICD-10: I60,61,63,64) or all-cause death. Secondary outcomes were non-fatal MACE, new onset of atrial fibrillation or flutter and effects on metabolic comorbidities. Non-fatal MACE was defined as hospitalization for acute coronary syndrome (ICD-10: I20.0, I21-22) or cerebrovascular event (ICD-10: I60,61,63,64). New onset of atrial fibrillation or flutter was defined as hospitalization or treatment in a specialized outpatient clinic with a diagnosed atrial fibrillation or flutter (ICD-10: I48). Remission of metabolic comorbidities at 2 years after surgery was defined as being without medication for diabetes (ATC: A10), dyslipidaemia (ATC: C10) or hypertension (ATC: C02,03,07,08 or 09) for individuals with these conditions before intervention, during a time frame of ±6 months (from 18 to 30 months) after the intervention.

### Statistics

Continuous variables are presented as means ± standard deviations or medians with interquartile range (IQR) as appropriate. Categorical variables are presented as numbers and proportions (%). The risk for primary and secondary outcomes were evaluated using a multivariable Cox regression model including all matching variables (age, sex, year of intervention, county of residence, disposable income, highest level of education, cardiovascular comorbidity, COPD, dyslipidaemia, number of diabetes drugs, insulin treatment, and duration of diabetes) with Hazard ratios (HR) and 95% confidence intervals (95%CI) as measures of association. Patients were followed until emigration, mortality, MACE or December 31, 2019 whichever came first. Time to event was estimated and visualized using the Kaplan-Meier method and presented as disease-free survival. Effects on metabolic comorbidities were evaluated using multivariable logistic regression (including all matching variables) for patients available for follow-up at this time point.

SPSS version 27 (IBM, Armonk, NY, USA), and R version 4.1.3 (matchit package, R Foundation for Statistical Computing, Vienna, Austria).

## Results

From the original cohort of 69,997 patients who underwent a primary gastric bypass or sleeve gastrectomy from January 1, 2007 until December 31, 2019 and 699,970 persons from the general population, 8659 patients from the surgical cohort with T2D and at least one drug as treatment, and 2879 non-surgical patients with T2D and GLP-1 agonist as part of treatment were identified. Of these, we were able to match 2161 patients (obesity class I (10.2%), class II (40.3%), class III (49.5%)) to 2161 non-surgical patients with an acceptable balance (Table 1). The most commonly used GLP-1 agonist in the non-surgical group was liraglutide (*n* = 1766; 81.7%), followed by dulaglutide (*n* = 213; 9.9%), exenatide (*n* = 130; 6.0%), lixisenatide (*n* = 34; 1.6%) and semaglutide (*n* = 18; 0.8%).

**Table 1:** Baseline characteristics after matching.

	Surgical group	GLP-1A group	Standardized mean difference
*n*	2161	2161	
Age	51.1 ± 9.29	51.5 ± 8.92	0.048
BMI	40.8 ± 5.49	n/a	
Sex			0.009
Men	760 (35.2%)	769 (35.6%)	
Women	1401 (64.8%)	1392 (64.4%)	
Education			0.016
Primary	393 (18.2%)	378 (17.5%)	
Secondary	1198 (55.4%)	1205 (55.8%)	
Higher	570 (26.4%)	578 (26.7%)	
Disposable income			0.045
Q1	545 (25.2%)	589 (27.3%)	
Q2	598 (27.7%)	619 (28.6%)	
Q3	569 (26.3%)	501 (23.2%)	
Q4	449 (20.8%)	452 (20.9%)	
Cardiovascular comorbidity	280 (13.0%)	285 (13.2%)	0.007
Dyslipidaemia	1258 (58.2%)	1275 (59.0%)	0.016
COPD	103 (4.8%)	108 (5.0%)	0.010
Surgical method			
Sleeve gastrectomy	413 (19.1%)	n/a	
Gastric bypass	1748 (80.9%)	n/a	
Number of diabetes drugs	2.5 ± 0.89	2.6 ± 0.87	0.098
Insulin	968 (44.2%)	925 (42.8%)	0.040
Diabetes duration	6.0 ± 4.15	6.0 ± 4.51	0.007

*n* number, *BMI* body mass index, *Qi* quartile, *COPD* chronic obstructive pulmonary disorder, *GLP-1A* Glucagon-like Peptide-1 receptor agonist.

During the study period, 54 patients (2.5%) and 52 non-surgical patients (2.4%) died, and 13 patients (0.6%) and 1 non-surgical patient (0.0%) emigrated resulting in a median follow-up of 4.2 (2.22–6.26) years in the surgical group and 3.9 (1.93–6.86) years in the non-surgical group.

### Major adverse cardiovascular events

**Fig. 1:**
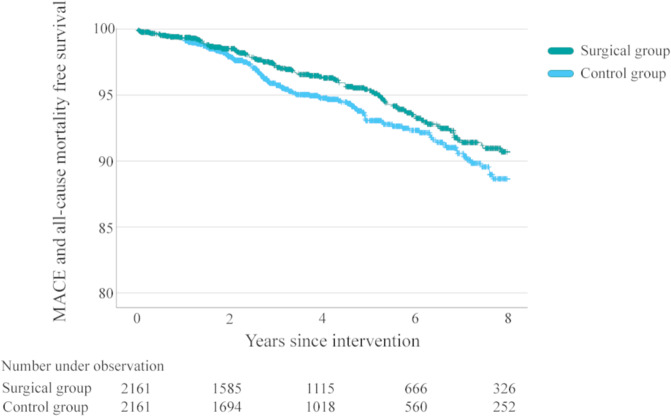
Major adverse cardiovascular events. Freedom of major adverse cardiovascular events (MACE) or all-cause mortality up to 8 years after initiation of GLP-1 receptor agonists or metabolic or bariatric surgery in persons with type 2 diabetes.

During the study period 113 patients in the surgical group suffered from MACE or mortality (cumulative incidence 9.3%; HR adjusted 0.76 (95%CI 0.59–0.98), *p* = 0.034) compared to 130 in the non-surgical group (cumulative incidence 11.3%) (Fig. 1).

### Non-fatal MACE, new onset of atrial fibrillation or flutter or heart failure

**Fig. 2:**
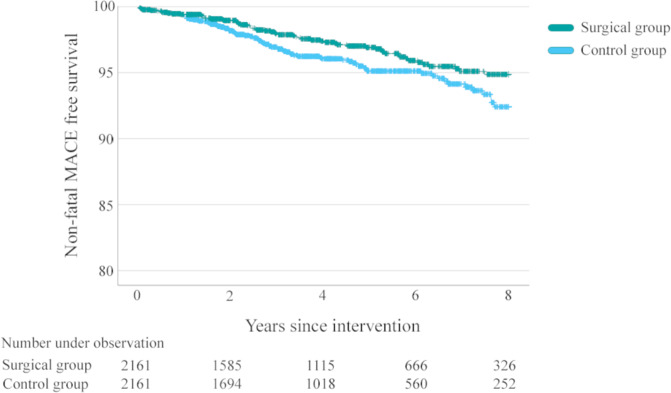
Non-fatal major cardiovascular events. Freedom of non-fatal major adverse cardiovascular events (MACE) up to 8 years after initiation of GLP-1 receptor agonists or metabolic and bariatric surgery in persons with type 2 diabetes.

During the study period 69 patients in the surgical group suffered from non-fatal MACE (8-year cumulative incidence 5.1%, HR adjusted 0.68 (95%CI 0.49–0.93), *p* = 0.015) compared to 92 patients in the non-surgical group (8-year cumulative incidence 7.6%) (Fig. 2). Among surgical patients and non-surgical without cardiovascular comorbidity at baseline, 23 patients in the surgical group developed atrial fibrillation or flutter (8-year cumulative incidence 2.2%) compared to 33 patients (8-year cumulative incidence 3.5%) in the non-surgical group (HR adjusted 0.69 (95%CI 0.39–1.21), *p* = 0.195), and new onset of heart failure occurred for 24 patients (8-year cumulative incidence 2.3%) in the surgical group compared to 34 patients (8-year cumulative incidence 3.5%) in the non-surgical group (HR adjusted 0.61 (95%CI 0.35–1.05), *p* = 0.077).

### Remission of metabolic co-morbidities

Of those eligible for follow-up, 2 years after the intervention, remission of dyslipidaemia was seen in 436 patients in the surgical group (45.0%) compared to 97 non-surgical patients (9.7%, *p* < 0.001). Of patients with pharmacological treatment for hypertension at baseline, 339 out of 1290 patients in the surgical group (26.3%) and 41 out of 1270 non-surgical patients (3.2%, *p* < 0.001) were not pharmacologically treated for hypertension 2 years after surgery. Of patients with a diagnosis of T2D, 843 (51.1%) were in remission after surgery (Table 2).

**Table 2:** Metabolic outcomes at 2 years after intervention.

	Surgical group	GLP-1A group	*P*
Numbers in remission			
Dyslipidaemia	546 (45.0%)	97 (9.7%)	<0.001
Hypertension	339 (26.0%)	41 (3.2%)	<0.001
Type-2 diabetes	843 (51.1%)	0 (0.0%)	<0.001
Numbers of diabetes drugs^a^	1.4 ± 0.65	2.4 ± 0.85	<0.001

^a^Mean number of diabetes drugs at 2 years after surgery (±standard deviation) for patients who did not experience remission of disease.

*GLP-1A* glucagon-like peptide-1 receptor agonist.

### Surgical outcomes

In the surgical group, 2130 (98.6%) had a registered follow-up including postoperative complications, of these 209 patients (9.8%) suffered from a postoperative complication of which 79 (3.8%) were considered to be a serious complication (Table 3). The cumulative event-free survival from MACE, all-cause mortality and serious postoperative surgical complications demonstrates a benefit of MBS (Supplementary Fig. 1). There were no deaths within 30 days after surgery. At 2 years after surgery, mean BMI-loss was 10.6 ± 4.03 kg/m^2^, with total weight-loss of 25.9 ± 8.55% and Excess BMI-loss 71.0 ± 25.81%.

**Table 3:** Postoperative complications at day 30 in the surgical group.

	Surgical group
Length of hospital stay, median (IQR), days	1 (1–2)
Follow-up at day 30, *n* (%)	2130 (98.6%)
Postoperative complication, *n* (%)	208 (9.8%)
Leak/deep intraabdominal infection, *n* (%)	42 (2.0%)
Bleeding, *n* (%)	52 (2.4%)
Wound complication, *n* (%)	34 (1.6%)
Bowel obstruction/stricture/ileus, *n* (%)	22 (1.0%)
Marginal ulcer, *n* (%)	10 (0.5%)
Cardiovascular complication, *n* (%)	6 (0.3%)
Pulmonary complication, *n* (%)	11 (0.5%)
Urinary tract infection, *n* (%)	8 (0.4%)
Venous thrombosis, *n* (%)	1 (0.1%)
Pain, *n* (%)	17 (0.8%)
Malnutrition/dehydration, *n* (%)	17 (0.8%)
Other complication, *n* (%)	28 (1.3%)
Serious postoperative complication, n (%)	79 (3.8%)

*IQR* interquartile range.

## Discussion

This paper demonstrates that the association between MACE and MBS in patients with severe obesity and T2D is reduced compared to patients with T2D that are treated with early GLP-1 RA. Surgery was associated with a very low mortality and postoperative complications in agreement with previous published results [20]. MBS was associated with significant improvements in risk factors for MACE and CVD such as remission of T2D, hypertension and treatment of dyslipidaemia.

Recently GLP-1 RA treatment has been suggested to be able to bridge the gap between conventional treatment and MBS in the light that semaglutide treatment has been shown to reduce weight by a mean of 14.9 % in patients with a mean baseline BMI of 37.9 ± 6.6 kg/m^2^ during a 68-week study period with 4.5% of patients discontinuing the treatment due to adverse events [21]. Liraglutide has been found to be less effective in achieving weight loss (an additional 5.4% compared to placebo) over 56 weeks [22]. Patients treated with MBS in the present study had a mean total weight-loss of 25.9% over after 2 years with a mean BMI of 40.8 kg/m^2^ at baseline. Since the control group was selected from the general public, data regarding change in weight is not available which is a limitation.

MBS was associated with a significantly greater chance of remission of hypertension and dyslipidaemia than control subjects treated with GLP-1 RA. The rate of remission of hypertension was lower than that seen in the only randomized controlled trial of MBS on hypertension (26 vs 51%) [6]. This difference may be related to the definition of remission. Semaglutide has been found to reduce both systemic (5.6 mmHg) and diastolic (3.0 mmHg) blood pressure compared to placebo [23]. In the present study, 51% of patients were found to be in remission of T2D at 2 years after surgery. No comparison to the non-surgical group is possible in light of the fact that patients in the non-surgical group had to be treated with GLP-1 RA. However, the STAMPEDE trial that randomized patients with severe obesity and T2D to MBS best medical treatment found that after 5 years the mean glycylated hemoglobin levels were 8.5% in the medical group and 7.3% after gastric bypass [5]. This indicates that MBS might have a better effect on T2D than medical treatment alone; however, mean glycylated hemoglobin levels after liraglutide treatment have been reported to be 7.1% [24].

Both liraglutide and semaglutide have been found to reduce the risk of MACE in patients with T2D and a high risk for CVD compared to placebo. The risk of MACE (death due to CVD, non-fatal MI, non-fatal stroke) during liraglutide was 13% lower than in the placebo group (HR) 0.87 (95%CI 0.78–0.97) [25]. Similar results were found for semaglutide risk of MACE (death due to CVD, non-fatal MI, non-fatal stroke) HR 0.74 (95%CI 0.58–0.95) compared to placebo [26]. This can be compared to the reduced risk for MACE seen in this study after MBS compared to treatment with GLP-1 RA in patients with T2D (HR) adjusted 0.76 (95%CI 0.59–0.98). Thus, MBS infers the same risk reduction compared to GLP-1 RA, that GLP-1 RA has been shown to have compared to placebo in patients with T2D.

When comparing surgical treatment to non-surgical treatment surgical complications need to be accounted for. In the surgical group 3.8% of patients suffered a serious complication. After the complication has resolved patients often have none or minor residual problems and the cumulative event-free survival from MACE, all-cause mortality and serious postoperative surgical complications favors MBS.

MBS has been shown to be associated with a decreased risk of heart failure and atrial fibrillation compared to non-surgical controls [2]. The risk of hospitalization for heart failure was not significantly different during treatment with liraglutide compared to controls in patients with T2D and CVD risk factors and a meta-analysis on the effect of GLP-1 RA found a non-statistical reduced trend toward a reduction in heart failure. Similarly, there is no effect of GLP-1 treatment on the risk of new onset atrial fibrillation [11, 27]. In the present study we found no significant difference between MBS and GLP-1 RA treatment with regard to heart failure and atrial fibrillation.

Strengths of this study is the high quality and completeness of data, and careful matching of available covarying factors. However, the study used a non-randomized design, based on registry data. Therefore, the included variables were limited to those available in the registries and the results should not be considered as proof of causation. Further limitations are lack of information on weight and weight change in the non-surgical group. However, in 2021 59% of men 40–60 years of age had a BMI equal or greater than 30. For women the corresponding number was 65.7% (annual report 2021, Swedish National Diabetes registry (www.ndr.nu/#/arsrapport)). Furthermore, the majority of patients in the non-surgical group were prescribed liraglutide. Few patients were prescribed with the more effective weight reducing GLP-1 RA such as semaglutide. Therefore, our results represent a comparison with less contemporary GLP-1 RA. Finally, with known socioeconomic inequalities in the use of GLP-1 RA as treatment for T2D [28], the study group had higher socioeconomic status compared to the standard MBS group in Sweden [29], limiting generalizability to a wider group of patients with severe obesity and T2D.

In summary, in this matched cohort study, MBS was associated with lower risk for MACE compared to treatment with early GLP-1 RA in patients with T2D.

## Supplementary information

Suppl fig and titel

Suppl fig 1

## Data Availability

Data cannot be shared publicly because of patient confidentiality under current Swedish legislation. Data are available from the Scandinavian Obesity Surgery Registry (contact via soreg@regionorebrolan.se), the Swedish Board of Health and Welfare (contact via Registerservice@socialstyrelsen.se) and Statistics Sweden (contact via mikrodata@scb.se) for researchers who meet the criteria for access to confidential data.
