# Supplementary material for: Major adverse cardiovascular events among patients with type-2 diabetes, a nationwide cohort study comparing primary metabolic and bariatric surgery to GLP-1 receptor agonist treatment
Source: Int J Obes (Lond). 2023 Jan 20;47(4):251–6. doi: 10.1038/s41366-023-01254-z (PMC10113141; doi:10.1038/s41366-023-01254-z)

**Supplementary Figure 1**

Cumulative event-free survival from MACE, all-cause mortality and serious postoperative complications for patients undergoing metabolic and bariatric surgery compared to a matched non-surgical group receiving GLP-1 agonists.


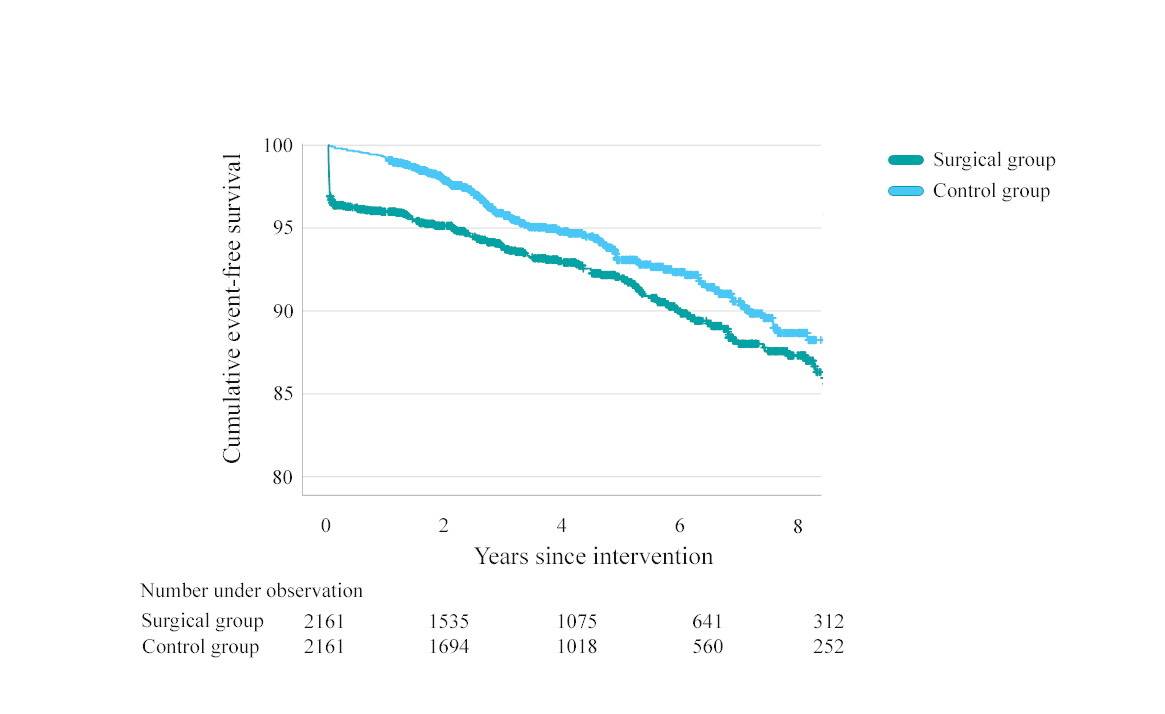

Supplement: Supplementary file 1 — Suppl fig and titel [file 41366_2023_1254_MOESM1_ESM.docx]
