# Supplementary figures and images for: Major adverse cardiovascular events among patients with type-2 diabetes, a nationwide cohort study comparing primary metabolic and bariatric surgery to GLP-1 receptor agonist treatment
Source: Int J Obes (Lond). 2023 Jan 20;47(4):251–6. doi: 10.1038/s41366-023-01254-z (PMC10113141; doi:10.1038/s41366-023-01254-z)

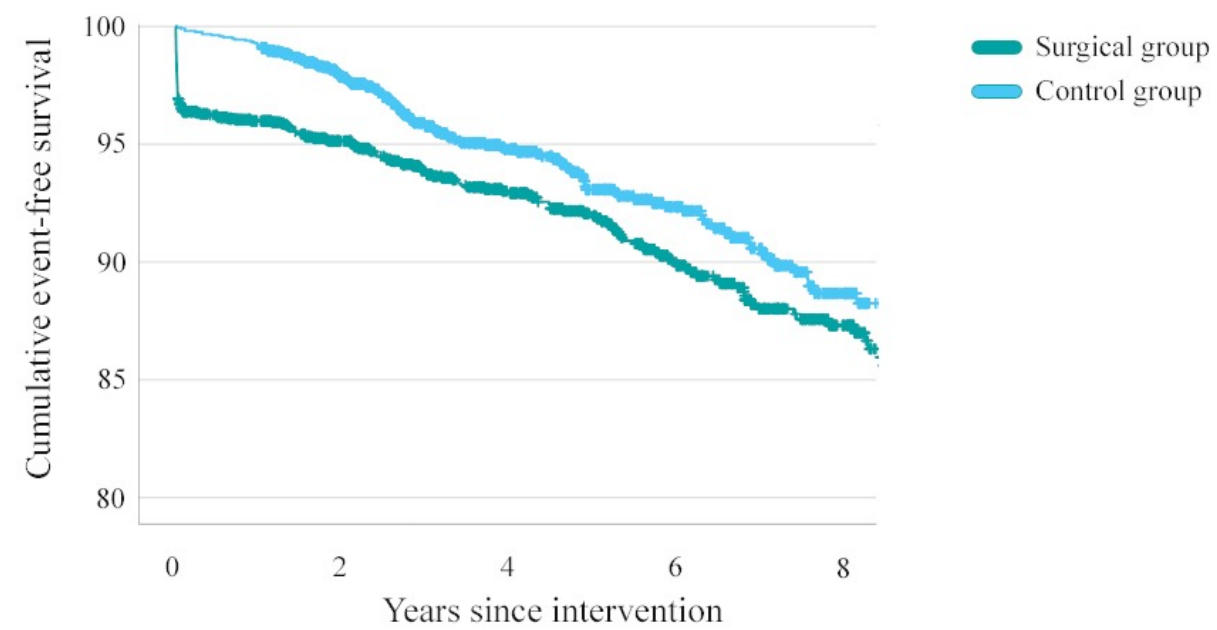

Number under observation

|                |      |      |      |     |     |
|----------------|------|------|------|-----|-----|
| Surgical group | 2161 | 1535 | 1075 | 641 | 312 |
| Control group  | 2161 | 1694 | 1018 | 560 | 252 |

Supplement: Supplementary file 2 — Suppl fig 1 [file 41366_2023_1254_MOESM2_ESM.pdf]
